# Machine learning integration of routine inflammatory biomarkers for predicting remote punctate ischemic lesions following intracerebral hemorrhage: a single-center retrospective study

**DOI:** 10.3389/fneur.2026.1851182

**Published:** 2026-06-29

**Authors:** Yibo Dong, Longyun Yi, Hongbo Tu

**Affiliations:** 1 Chongqing Orthopedic Hospital of Traditional Chinese Medicine, Chongqing, China; 2 Honghui Hospital, Xi'an Jiaotong University, Xi'an, Shaanxi, China

**Keywords:** cerebral infarction, intracerebral hemorrhage, machine learning, nomogram, systemic immune-inflammation index, XGBoost

## Abstract

**Background:**

Remote Punctate Ischemic Lesions (RPIL) occurring during the acute phase of intracerebral hemorrhage (ICH) represent a paradox of “ischemia within hemorrhage” that is associated with worse functional outcome. While the concept of “thrombo-inflammation” is gaining traction, the predictive utility of novel biomarkers like the Systemic Immune-Inflammation Index (SII) combined with advanced machine learning (ML) remains uncharacterized.

**Methods:**

In this retrospective cohort study conducted between January 2019 and December 2025, 12,327 patients with ICH were initially screened, and 6,134 were included after strict exclusion criteria. The cohort was randomly split into training (*n* = 4,294) and validation (*n* = 1,840) sets. Feature selection was performed using LASSO regression. We benchmarked 15 ML algorithms, ranging from Logistic Regression to ensemble methods (XGBoost, Random Forest). Model interpretability was achieved via feature importance analysis and a clinical nomogram.

**Results:**

LASSO regression identified six key predictors: Age, History of Diabetes, SII, D-Dimer, Glucose, and Fibrinogen. In the ML benchmark, XGBoost achieved the highest discrimination (AUC = 0.799), outperforming the Neural Network (AUC = 0.798) and standard Logistic Regression (AUC = 0.770). The derived nomogram demonstrated excellent calibration (Mean Absolute Error = 0.034) and clinical net benefit in Decision Curve Analysis (DCA). Crucially, inflammatory and coagulation markers (SII, Fibrinogen) were identified as top-tier predictors, corroborating the immuno-thrombotic mechanism.

**Conclusion:**

We present a robust ML framework demonstrating that systemic inflammation and hypercoagulability are strongly associated with post-ICH ischemia. The XGBoost model offers precision, while the nomogram provides translational utility for bedside risk stratification. However, as this study relies on single-center data, future multicenter external validation is imperative to confirm the generalizability and clinical applicability of these models before broad implementation.

## 1 Introduction

Intracerebral hemorrhage (ICH) constitutes approximately 10–15% of all strokes but disproportionately contributes to global stroke mortality and disability (1). While acute management traditionally focuses on halting hematoma expansion and reducing intracranial pressure, a paradoxical and severe complication—the development of Remote Punctate Ischemic Lesions (RPIL) during hospitalization—is increasingly recognized. Recent large-scale epidemiological data indicate that 3–12% of ICH patients develop remote ischemic lesions, significantly worsening functional outcomes (2, 3). According to the latest Global Burden of Disease (GBD) study (2024), stroke remains a predominant cause of global morbidity, with hemorrhagic stroke disproportionately contributing to severe long-term disability and fatal complications (4).

The pathophysiology underlying this “hemorrhage-ischemia paradox” involves a complex cascade where early hematoma degradation triggers a profound systemic inflammatory response. This inflammation is inextricably linked with the activation of coagulation cascades (a process termed immunothrombosis) and acute metabolic stress, such as stress hyperglycemia. Specifically, the initial hemorrhagic injury triggers a systemic inflammatory response, releasing neutrophils and cytokines that can activate the coagulation cascade (5). Routine clinical biomarkers capture different axes of this thrombo-inflammatory network. For instance, D-dimer and fibrinogen reflect hypercoagulability, while fasting glucose represents metabolic stress. Furthermore, composite hematological biomarkers, such as the Systemic Immune-Inflammation Index (SII) (integrating platelet, neutrophil, and lymphocyte counts), have proven effective in predicting outcomes in ischemic stroke (6). However, the exact predictive value of SII and other composite markers for post-ICH ischemic events remains insufficiently established.

Predicting post-ICH RPIL is challenging because the interplay among systemic inflammation, coagulation activation, and metabolic stress is highly heterogeneous. Traditional statistical methods (e.g., logistic regression) generally assume linear relationships and may fail to capture the non-linear interactions between metabolic, inflammatory, and coagulation factors in this specific disease context. Advanced machine learning (ML) algorithms, such as Extreme Gradient Boosting (XGBoost), have demonstrated superior performance in medical prognostication by modeling high-dimensional data (7, 8). ML models are exceptionally adept at identifying intricate non-linear relationships and higher-order interactions among diverse clinical variables, making them uniquely suited for predicting secondary ischemic events after acute ICH.

Therefore, this study aims to: (1) investigate the predictive value of routine laboratory biomarkers (including SII, SIRI, D-dimer, and glucose) for post-ICH RPIL; (2) benchmark 15 different ML algorithms to identify the optimal predictive model; and (3) develop a clinically interpretable nomogram to guide personalized monitoring and risk stratification.

## 2 Materials and methods

### 2.1 Study design and population

This retrospective study analyzed data from patients diagnosed with spontaneous ICH at Xi’an Honghui Hospital between January 1,2019 to December 31,2025. The raw dataset initially contained 12,327 patients.

**Figure 1:**
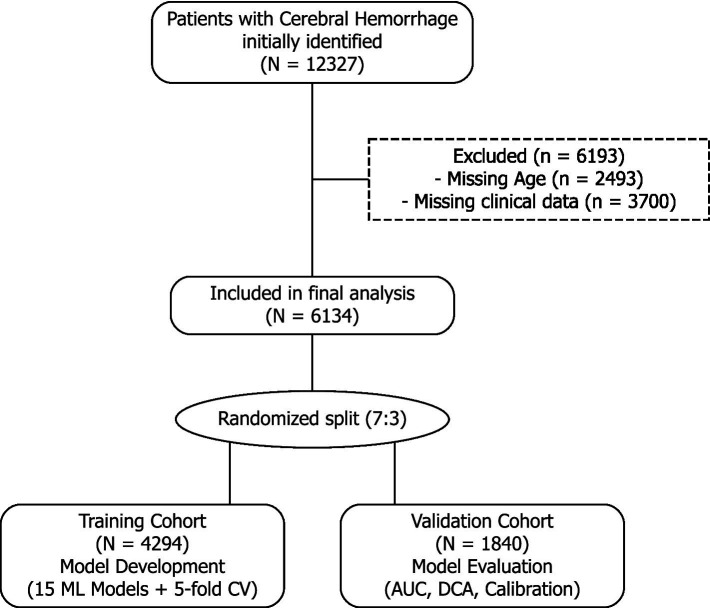
Flowchart of patient selection and study design. The study initially identified 12,327 patients with intracerebral hemorrhage (ICH). After applying exclusion criteria (missing age or >20% missing clinical data), 6,134 patients were included in the final analysis and randomly divided into a training cohort (70%) and a validation cohort (30%). ICH, intracerebral hemorrhage; ML, machine learning; CV, cross-validation.

Exclusion criteria were: (1) missing age data (*n* = 2,493); (2) significant missingness (>20%) in key clinical variables (*n* = 3,700). A final cohort of 6,134 patients was included (Figure 1). The dataset was randomly divided into a training set (70%, *n* = 4,294) and a validation set (30%, *n* = 1,840).

### 2.2 Data collection and definitions

Clinical characteristics included demographics (Age, Sex), medical history (Hypertension, Diabetes, Smoking), and admission laboratory indicators. The inflammatory indices were calculated as follows: SII = (Platelet count × Neutrophil count)/Lymphocyte count. SIRI = (Neutrophil count × Monocyte count)/Lymphocyte count. The primary outcome was incident cerebral infarction (post-ICH RPIL), strictly defined as a newly developed ischemic lesion occurring within 14 days of admission for the index ICH. Given the retrospective nature of this study, standardized DWI screening was not universally performed for all patients. Instead, neuroimaging was typically acquired upon clinical deterioration or as part of routine follow-up. To rigorously ascertain the outcome and distinguish incident infarcts from chronic ischemic lesions, all available imaging data (MRI or CT) were retrospectively reviewed by two independent, blinded neurologists. Incident RPIL was radiologically confirmed if there was restricted diffusion on DWI or a distinctly new, well-demarcated hypodense lesion on follow-up CT that was not present on the admission baseline scan. Discrepancies were resolved by consensus or by a third senior radiologist.

### 2.3 Data preprocessing and missing value imputation

Initially, 12,327 patients were screened. After strict application of the exclusion criteria, 6,193 patients were excluded. This exclusion was primarily due to missing fundamental demographic data (e.g., age) stemming from undocumented emergency admissions or retrospective extraction errors from the legacy Electronic Health Record system, as well as significant missingness (defined as missing values in >20% of the predictor variables) or missing primary outcome data. To assess potential selection bias, a comparison of available baseline variables between the included cohort (*n* = 6,134) and the excluded cohort (*n* = 6,193) was performed (Supplementary Table S2). Although minor statistical differences were observed in the prevalence of hypertension and diabetes due to the extremely large sample size, the overall clinical distributions remained highly comparable, indicating that the exclusion process did not introduce severe systematic selection bias.

For the included population, residual missing values were handled using the median imputation method. This approach was selected specifically due to the non-normal distribution and potential skewness inherent in clinical laboratory parameters (e.g., D-dimer, SII, and white blood cell counts). Unlike mean imputation, median imputation is robust against outliers and preserves the original distribution characteristics of the biomedical data. Crucially, to rigorously prevent data leakage, imputation parameters (e.g., median values) were derived exclusively from the training set and subsequently applied to both the training and validation sets after the initial data split. To address potential biases introduced by single median imputation, we additionally performed a sensitivity analysis using Multiple Imputation by Chained Equations (MICE) to verify the robustness of our feature selection and model performance.

All preprocessing procedures were performed using the caret package in R (version 4.5.2) prior to model training. Our rigorous approach to evaluate the robustness of the missing data handling strategy utilized Multiple Imputation by Chained Equations (MICE). This sensitivity analysis adheres to contemporary epidemiological reporting standards, which strongly caution against single-imputation methods that artificially deflate variance and disrupt the underlying covariance structure in machine learning pipelines (9, 10). Specifically, five independent imputed datasets (m = 5) were generated utilizing the Predictive Mean Matching (PMM) algorithm. The primary predictive model (XGBoost) was subsequently re-trained and re-evaluated on each of these five datasets to strictly assess the stability of its predictive performance.

### 2.4 Statistical analysis and machine learning

**Figure 2:**
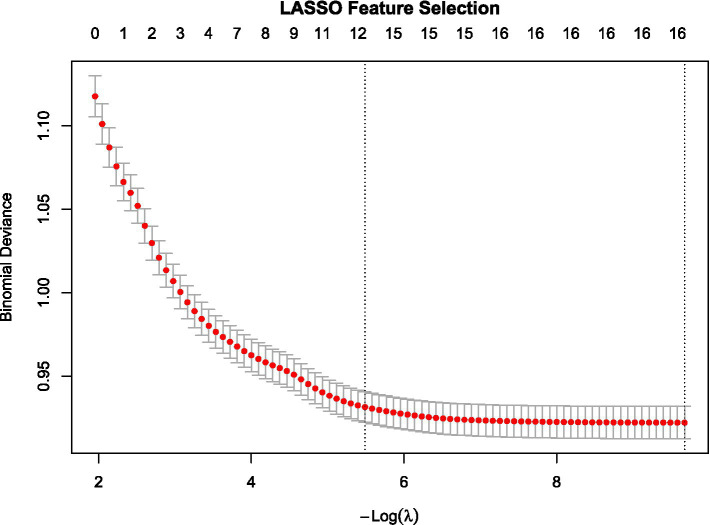
Feature selection using the Least Absolute Shrinkage and Selection Operator (LASSO) binary logistic regression model. **(A)** Tuning parameter (*λ*) selection in the LASSO model using 10-fold cross-validation via minimum criteria. The partial likelihood deviance is plotted against log(λ). Vertical dotted lines indicate the optimal values (λ.min and λ.1se). **(B)** LASSO coefficient profiles of the clinical features. Six features with non-zero coefficients were selected at the optimal λ.

First, to ensure bedside simplicity and high clinical interpretability, Least Absolute Shrinkage and Selection Operator (LASSO) regression was utilized to aggressively shrink coefficients and identify a sparse subset of the most crucial non-zero predictors. These strictly selected six features (Age, DM, SII, D-dimer, Glucose, and Fibrinogen) were exclusively used to construct the traditional Logistic regression-based Nomogram. Second, recognizing that traditional linear models and strict feature selection may discard valuable nuanced data, we employed a full-feature modeling approach for the machine learning algorithms. Specifically, the XGBoost model incorporated the full set of collected variables (that passed initial collinearity diagnostics, thus retaining markers like NLR, PLR, etc.) to fully exploit non-linear interactions and maximize absolute predictive discrimination. The prior application of LASSO penalty is particularly crucial in this context. Expert statistical recommendations strongly advise against traditional stepwise selection methods in high-dimensional biological datasets due to instability and inflated false-positive rates. LASSO gracefully resolves these issues by shrinking coefficients of highly collinear inflammatory indices (e.g., handling the inherent collinearity among SII, NLR, and PLR), yielding a more reproducible and parsimonious predictive subset (11). To rigorously prevent model overfitting while handling high-dimensional, collinear biomarker data, feature selection was performed using LASSO regression with 10-fold cross-validation (Figure 2). This regularization technique aligns with the latest methodological guidelines for developing robust and parsimonious clinical prediction models, ensuring that only the most informative features are retained (12, 13). Furthermore, the combination of LASSO feature selection and nomogram visualization has been recently validated as a highly effective framework for predicting other severe secondary complications of ICH, such as early acute hydrocephalus (14). We trained and validated 15 supervised ML algorithms: Logistic Regression (GLM), LDA, Naive Bayes, Decision Tree, KNN, Random Forest, SVM (Linear/Radial), Neural Network, GBM, AdaBoost, Bagged Trees, C5.0, and XGBoost (15). Hyperparameters were optimized using grid search within the caret framework in R (version 4.5.2). Performance was evaluated using the Area Under the Curve (AUC). A Nomogram was constructed based on the logistic model. Beyond traditional discriminative metrics (e.g., AUC), we strictly utilized Decision Curve Analysis (DCA) to quantify the net clinical benefit of the predictive models across various threshold probabilities, aligning with standard consensus on evaluating the real-world utility of diagnostic tools prior to clinical implementation (16).

Furthermore, the development, validation, and transparent reporting of our machine learning prediction models and clinical nomogram strictly adhered to the newly updated TRIPOD+AI (Transparent Reporting of a multivariable prediction model of Individual Prognosis Or Diagnosis Including AI) consensus statement published in 2024 (15).

## 3 Results

### 3.1 Baseline characteristics of the study population

A total of 6,134 patients with spontaneous ICH were included in the final analysis, among whom 4,618 (75.3%) did not develop cerebral infarction (No RPIL group) and 1,516 (24.7%) experienced incident cerebral infarction (Post-ICH RPIL group) during hospitalization. The baseline demographic, clinical, and laboratory characteristics of the overall cohort stratified by RPIL occurrence are detailed in Table 1. Compared to the No RPIL group, patients who developed post-ICH RPIL were significantly older (67.52 ± 43.53 vs. 59.63 ± 16.22 years, *p* < 0.001) and had higher prevalences of hypertension (54.6% vs. 46.1%, *p* < 0.001) and diabetes mellitus (17.1% vs. 6.0%, *p* < 0.001). Furthermore, the Post-ICH RPIL group exhibited significantly elevated levels of systemic inflammatory and coagulation markers, including WBC, D-dimer, fibrinogen, SII, SIRI, NLR, and PLR (all *p* < 0.001), indicating a profound state of systemic inflammation and hypercoagulability.

**Table 1:** Baseline clinical and laboratory characteristics of the study population.

Variables	Total cohort (*n* = 6,134)	No CI (*n* = 1,516)	Post-ICH CI (*n* = 4,618)	*p*-value
Demographics				
Age, years (mean ± SD)	65.78 ± 39.30	59.63 ± 16.22	67.52 ± 43.53	**<0.001**
Male sex, *n* (%)	3,529 (57.5%)	930 (61.3%)	2,599 (56.3%)	**0.001**
Comorbidities and risk factors				
Hypertension, *n* (%)	3,195 (52.5%)	690 (46.1%)	2,505 (54.6%)	**<0.001**
Diabetes mellitus, *n* (%)	875 (14.4%)	90 (6.0%)	785 (17.1%)	**<0.001**
Smoking, *n* (%)	2,100 (48.5%)	547 (51.4%)	1,553 (47.6%)	**0.034**
Laboratory findings				
Fasting glucose, mmol/L (mean ± SD)	7.78 ± 3.65	8.15 ± 3.65	7.65 ± 3.65	**<0.001**
D-dimer, mg/L (median [IQR])	0.33 [0.22, 0.84]	0.55 [0.27, 2.24]	0.29 [0.20, 0.57]	**<0.001**
Fibrinogen, g/L (mean ± SD)	3.29 ± 1.11	2.91 ± 1.11	3.41 ± 1.08	**<0.001**
White blood cell, 10*9/L (mean ± SD)	8.48 ± 3.95	10.63 ± 4.95	7.78 ± 3.27	**<0.001**
Neutrophil, 10*9/L (mean ± SD)	6.45 ± 3.86	8.66 ± 4.79	5.72 ± 3.18	**<0.001**
Lymphocyte, 10*9/L (mean ± SD)	1.33 ± 0.66	1.23 ± 0.72	1.36 ± 0.64	**<0.001**
Monocyte, 10*9/L (mean ± SD)	0.52 ± 0.27	0.58 ± 0.33	0.50 ± 0.24	**<0.001**
Platelet,10*9/L (Mean ± SD)	205.97 ± 76.66	198.83 ± 82.15	208.31 ± 74.63	**<0.001**
Composite inflammatory indices				
SII (median [IQR])	849.87 [497.47, 1588.17]	1353.06 [713.07, 2377.27]	755.30 [465.98, 1316.09]	**<0.001**
SIRI (median [IQR])	1.95 [1.11, 3.94]	3.46 [1.69, 7.52]	1.68 [1.02, 3.12]	**<0.001**
NLR (median [IQR])	4.20 [2.65, 7.97]	7.12 [3.86, 12.67]	3.73 [2.47, 6.23]	**<0.001**
PLR (median [IQR])	159.31 [114.73, 225.34]	173.24 [119.69, 251.56]	155.17 [113.67, 217.91]	**<0.001**

### 3.2 Characteristics and feature selection

Prior to model development, the total cohort was randomly partitioned into a training cohort (*n* = 4,294) and a validation cohort (*n* = 1,840) at a 7:3 ratio. As shown in Supplementary Table S1, the baseline clinical and demographic characteristics were well-balanced between the training and validation sets, with no significant differences observed in key variables such as age, sex, hypertension, and essential laboratory indicators (all *p* > 0.05), ensuring the reliability of subsequent machine learning evaluations.

The overall incidence of RPIL in the cohort was 24.71%. Using LASSO regression, six variables with non-zero coefficients were selected from the high-dimensional dataset: Age, History of Diabetes, SII, D-Dimer, Glucose, and Fibrinogen. These features represent a convergence of metabolic (Diabetes, Glucose), inflammatory (SII), and coagulation (D-Dimer, Fibrinogen) pathways.

### 3.3 Nomogram construction and validation

**Figure 3:**
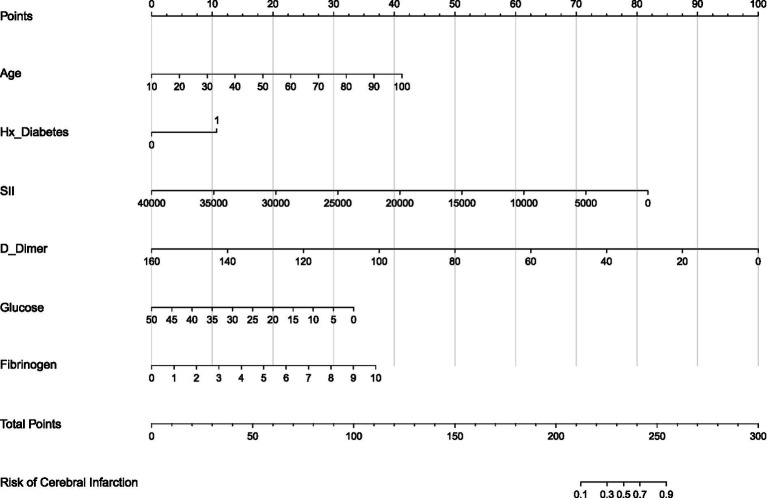
Diagnostic nomogram for predicting the risk of cerebral infarction in patients with intracerebral hemorrhage. The nomogram integrates six predictors: Age, History of Diabetes, SII, D-Dimer, Glucose, and Fibrinogen. Instructions: To use the nomogram, locate the patient’s value for each variable on its respective axis. Draw a vertical line upwards to the “Points” axis to determine the score. Sum the scores for all variables to obtain the “Total Points.” Finally, draw a vertical line downwards from the “Total Points” axis to the “Risk” axis to obtain the predicted probability of cerebral infarction. SII, Systemic Immune-Inflammation Index.

To facilitate clinical application, a nomogram was built (Figure 3).

#### 3.3.1 Discrimination

**Figure 4:**
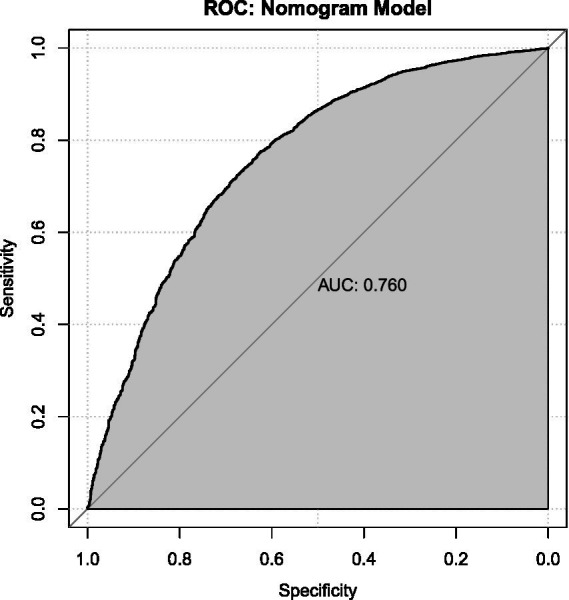
Receiver Operating Characteristic (ROC) curve of the nomogram in the validation cohort. The nomogram achieved an Area Under the Curve (AUC) of 0.760, indicating satisfactory discriminatory ability for predicting cerebral infarction. The diagonal line represents a random classifier (AUC = 0.5).

**Figure 5:**
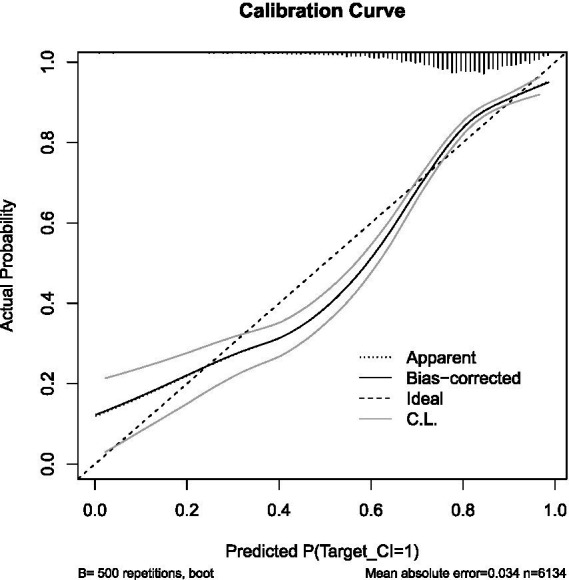
Calibration curve of the nomogram for predicting cerebral infarction. The x-axis represents the predicted probability of cerebral infarction, and the y-axis represents the actual observed probability. The diagonal gray dashed line represents perfect prediction by an ideal model. The solid blue line represents the performance of the nomogram, with a Mean Absolute Error (MAE) of 0.034, indicating excellent agreement between prediction and observation. B = 500 indicates bootstrap resampling times.

The nomogram achieved an AUC of 0.760 (Figure 4). While slightly lower than XGBoost, it offers superior interpretability. Calibration: The calibration plot (Figure 5) showed high agreement between predicted and observed risks (Mean Absolute Error = 0.034). We heavily emphasized this metric, as contemporary methodological consensus recognizes that proper calibration—ensuring that predicted probabilities accurately reflect true event rates—is the ‘Achilles heel’ of predictive analytics and is arguably more critical than discrimination for safe clinical decision-making (17).

#### 3.3.2 Decision curve analysis (DCA)

**Figure 6:**
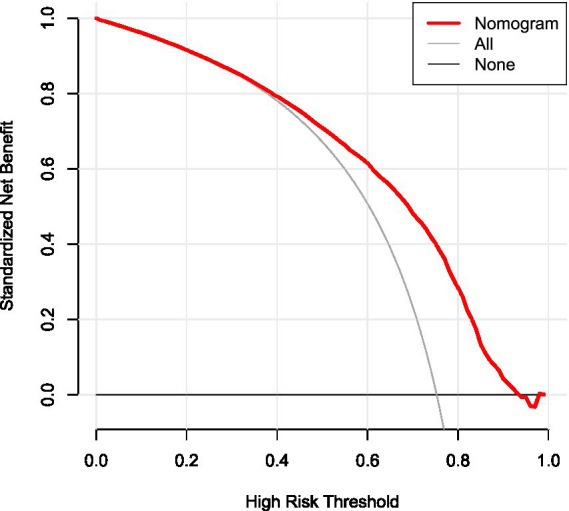
Decision Curve Analysis (DCA) of the nomogram. The y-axis measures the net benefit. The red line represents the nomogram model. The gray line assumes all patients have cerebral infarction (“Treat All”), and the black horizontal line assumes no patients have cerebral infarction (“Treat None”). The decision curve shows that using the nomogram to predict cerebral infarction adds more net benefit than the “treat-all” or “treat-none” strategies across a wide range of threshold probabilities (approx. 5 to 60%).

The DCA (Figure 6) demonstrated that using the nomogram provides a net benefit over “treat-all” or “treat-none” strategies across a threshold probability range of 5 to 60%.

### 3.4 Machine learning benchmark

**Figure 7:**
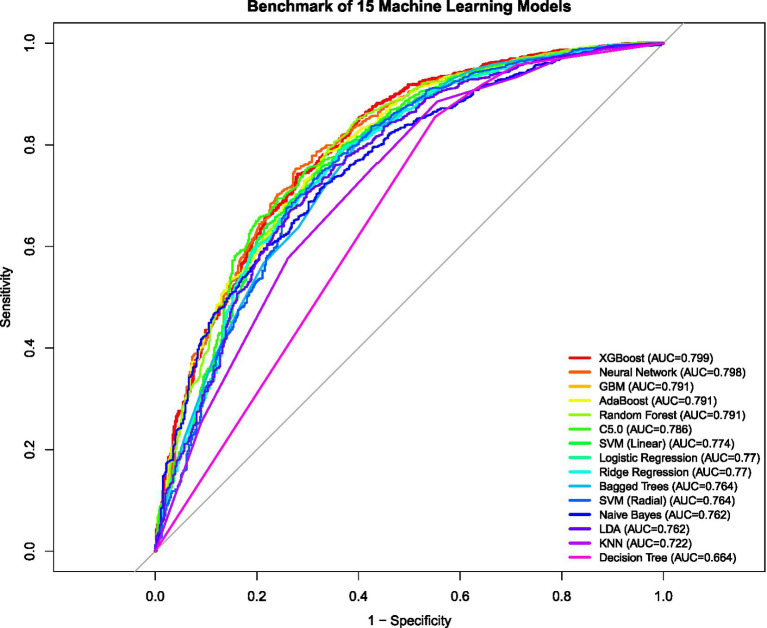
Benchmark comparison of 15 machine learning algorithms for predicting cerebral infarction. The Receiver Operating Characteristic (ROC) curves for all 15 models are displayed based on the validation cohort. XGBoost achieved the highest AUC (0.799), followed by Neural Network (0.798) and Random Forest (0.791), outperforming traditional methods like Logistic Regression (0.770) and Decision Tree (0.664). AUC, area under the curve; SVM, support vector machine; KNN, K-Nearest Neighbors; LDA, linear discriminant analysis.

The performance of 15 ML models is summarized in Figure 7.

#### 3.4.1 Top performer

XGBoost achieved the highest discrimination with an AUC of 0.799 (95% CI: 0.78–0.82) in the validation cohort.

#### 3.4.2 Runners-up

Neural Network (AUC = 0.798) and Random Forest (AUC = 0.791) also showed excellent performance.

#### 3.4.3 Traditional models

Logistic Regression performed robustly (AUC = 0.770) but was outperformed by the ensemble methods, highlighting the value of capturing non-linear relationships.

### 3.5 Sensitivity analysis of missing data imputation

To further validate the reliability of our findings against missing data variations, we evaluated the XGBoost model’s performance across the five datasets generated by the MICE method. The model demonstrated remarkable stability, achieving a mean Area Under the Curve (AUC) of 0.8358 with a marginal standard deviation of ±0.0054 (range: 0.8304 to 0.8424). This result not only confirms the extraordinary robustness of our modeling approach but also suggests that comprehensive multiple imputation actually unlocks further predictive potential compared to the primary median-imputed baseline model (detailed metrics are provided in Supplementary Table S1).

### 3.6 Feature importance

**Figure 8:**
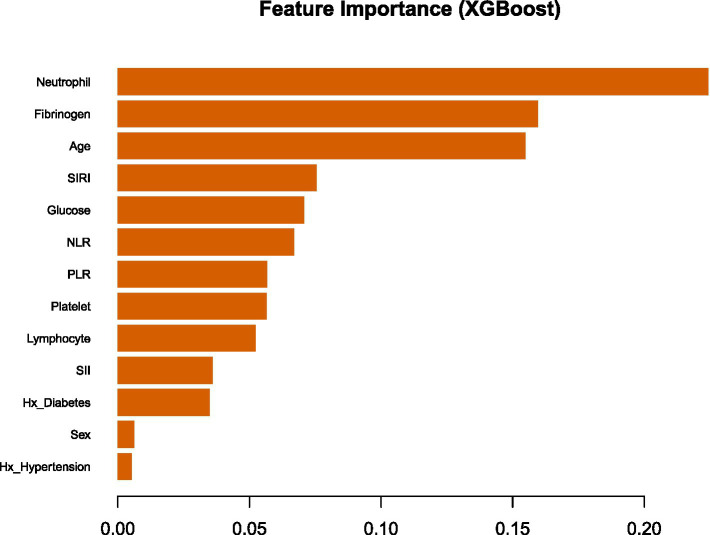
Feature importance ranking based on the XGBoost model. Unlike the nomogram which was restricted to the 6 features selected by LASSO regression for simplicity, the XGBoost model incorporated the full set of non-collinear variables to capture the complex, non-linear predictive weights of additional inflammatory indices such as NLR and PLR.

Analysis of the XGBoost model (Figure 8) revealed that Age was the most dominant predictor. Notably, Fibrinogen and Neutrophil counts (components of SII) ranked immediately after Age, confirming that systemic inflammation and hypercoagulability are the primary drivers of ischemic risk in this population.

## 4 Discussion

In this large-scale study of 6,134 ICH patients, we developed and validated a machine learning framework to predict the risk of concomitant cerebral infarction. Our findings underscore that XGBoost provides the highest predictive accuracy (AUC ~ 0.80), while a simplified Nomogram incorporating SII and Fibrinogen offers a practical bedside tool.

### 4.1 The mechanism: immuno-thrombosis

Our study is among the first to utilize machine learning to identify SII, Fibrinogen, and Glucose as top-tier predictors for Remote Punctate Ischemic Lesions (RPIL) following ICH. These findings provide compelling clinical evidence for the concept of “Immuno-thrombosis”—a pathological synergy where the immune system activates coagulation (5, 18). Following ICH, the breakdown of the blood–brain barrier and hematoma release trigger a massive inflammatory storm. Recent mechanistic studies by Chen et al. (19) and Wang et al. (20) have demonstrated that this neuroinflammatory response involves significant neutrophil infiltration and microglia activation, which exacerbates secondary brain injury and neuronal apoptosis (21). Our model suggests this process is not confined to the brain; the systemic elevation of neutrophils (reflected by a high SII) likely mirrors this central inflammation, creating a systemic pro-thrombotic state. Specifically, activated neutrophils release Neutrophil Extracellular Traps (NETs), which serve as scaffolds for platelet aggregation and fibrin deposition, promoting thrombosis even in a hemorrhagic state (18). This paradigm is strongly supported by recent comprehensive reviews (2024), which highlight that the intricate crosstalk between NETosis and the coagulation cascade is a central driver of immunothrombosis, determining the ultimate fate of the ischemic penumbra (22). The prominent predictive weight of the systemic immune-inflammation index (SII) in our model is strongly corroborated by recent neuroinflammatory research, which identifies SII not merely as a bystander, but as a highly sensitive surrogate marker for the massive release of damage-associated molecular patterns (DAMPs) and subsequent blood–brain barrier disruption following acute brain injury (23).

**Figure 9:**
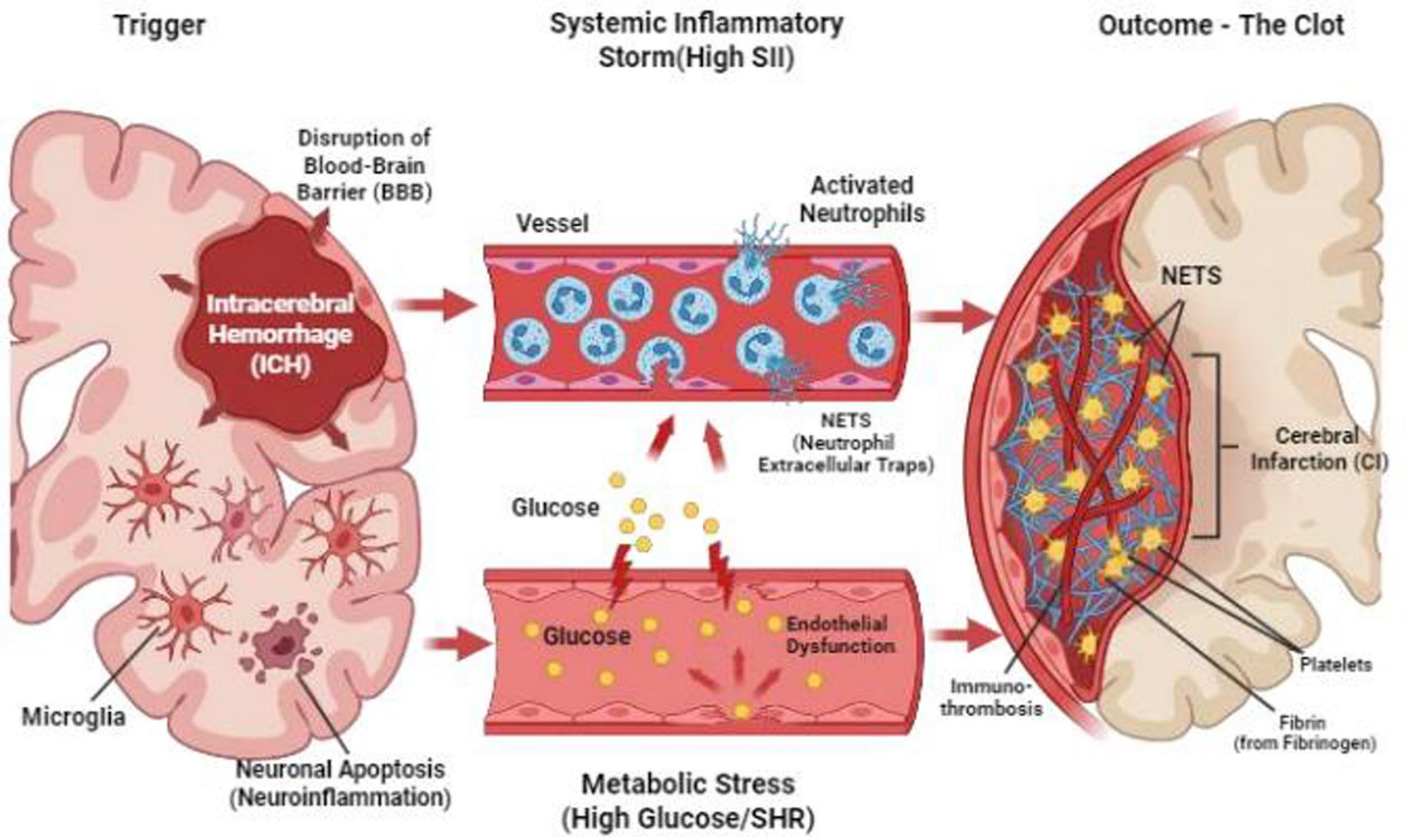
Proposed pathophysiological mechanism linking ICH to subsequent RPIL. ICH triggers systemic inflammation (high SII) and metabolic stress (high glucose). Activated neutrophils release NETs, which act as scaffolds for platelet aggregation and fibrin deposition (from fibrinogen), leading to immuno-thrombosis and secondary cerebral infarction. Created in BioRender. Dong, Y. (2026). https://BioRender.com/mb9pw8a.

Furthermore, the high predictive weight of Glucose in our XGBoost model highlights the critical role of metabolic stress. Hyperglycemia impairs endothelial function and amplifies the pro-inflammatory cytokine storm, forming a vicious “Metabolic-Inflammatory-Thrombotic” cycle that significantly elevates the risk of secondary ischemic events (Figure 9).

### 4.2 Machine learning vs. traditional scores

Traditional stroke risk scores (e.g., CHA2DS2-VASc) are designed for atrial fibrillation and do not account for the acute inflammatory storm of ICH (21, 24). Our comprehensive benchmark of 15 algorithms demonstrated that ensemble machine learning (ML) models, particularly XGBoost (AUC = 0.799) and Neural Networks (AUC = 0.798), consistently outperformed traditional logistic regression (AUC = 0.770) and single Decision Trees (AUC = 0.664). This superiority aligns with the findings of Chen et al. (25) in Frontiers in Neurology, who showed that integrating routine laboratory blood tests via ML significantly enhances mortality prediction in ICH patients compared to standard clinical scores (e.g., ICH Score). Traditional regression models assume linearity and additivity, often oversimplifying the complex biological interactions between inflammation, metabolism, and coagulation. In contrast, XGBoost utilizes gradient boosting to capture high-order non-linear relationships and interactions (e.g., the synergistic effect of Age × SII × Glucose), thereby reducing bias and improving generalization in heterogeneous real-world cohorts (3, 26).

Furthermore, the integration of continuous physiological variables (like Glucose and SII) into ML models offers a more granular risk assessment than categorical cut-offs. As highlighted by Bateman et al. in Critical Care, microvascular dysfunction and impaired oxygen-dependent ATP efflux are hallmarks of critical illness, leading to capillary flow maldistribution (27). Our ML models likely capture these subtle physiological perturbations reflected in routine blood markers—such as the metabolic stress indicated by hyperglycemia and the inflammatory rheology indicated by elevated Fibrinogen—which traditional scores might overlook. By modeling these continuous variables without artificial discretization, XGBoost preserves the “physiological signal” essential for predicting secondary ischemic events in the vulnerable post-ICH brain.

### 4.3 Clinical implications

The identification of a high-risk phenotype for RPIL (characterized by elevated SII, Fibrinogen, and Glucose) presents a significant therapeutic dilemma: preventing ischemia without exacerbating the initial hemorrhage. Our XGBoost model serves as a crucial “gatekeeper,” allowing clinicians to move beyond a “one-size-fits-all” approach toward precision medicine.

For patients identified as low-risk by our nomogram, standard supportive care (e.g., hydration, mechanical prophylaxis) is likely sufficient. Given the extreme risk of hematoma expansion in the acute phase of ICH, aggressive antithrombotic interventions are generally contraindicated. Therefore, for ICH patients identified as high-risk for secondary cerebral infarction by our XGBoost model or nomogram, clinicians should prioritize intensive non-invasive monitoring rather than immediate pharmacological prophylaxis. We recommend increasing the frequency of Transcranial Doppler (TCD) to monitor cerebral microemboli, conducting strict bedside neurological evaluations, and meticulously maintaining optimal cerebral perfusion pressure. The management strategy must delicately balance the prevention of ischemic events with the absolute priority of avoiding catastrophic re-bleeding.

Given the catastrophic risk of hematoma expansion, the use of any antithrombotic agents (even short-acting agents like tirofiban) in the acute phase of ICH remains highly controversial and potentially unsafe. Therefore, the primary utility of our model is to prompt intensive non-invasive monitoring (e.g., TCD microemboli monitoring) and stringent physiological optimization (e.g., blood pressure and glucose control) for high-risk patients. Any pharmacological “bridging strategy” for RPIL prevention in this vulnerable population remains purely speculative and must be strictly confined to rigorously designed, future randomized controlled trials. Additionally, given the strong predictive weight of Glucose, aggressive management of stress hyperglycemia should be prioritized to dampen the “metabolic-inflammatory” axis described in our mechanism.

### 4.4 Limitations and future directions

Despite the strengths of our study, including the large sample size (*N* = 6,134) and the robust benchmarking of 15 ML algorithms, several limitations must be acknowledged. First and foremost, the retrospective, single-center design may introduce selection bias. Because all data were derived from a single institution, the generalizability of our predictive models to independent, multi-center, or ethnically diverse populations remains unestablished. To mitigate this, we employed strict inclusion/exclusion criteria and performed rigorous internal validation using 5-fold cross-validation and bootstrapping, which demonstrated stable model performance. However, we strongly emphasize that future validation using large-scale, external, and multicenter cohorts is a mandatory next step to definitively confirm the robustness and clinical applicability of our proposed XGBoost model and nomogram.

Second, although we included comprehensive clinical and laboratory variables, unmeasured residual confounders may exist. A primary limitation of this study is the lack of detailed baseline neuroimaging data (e.g., hematoma volume, specific ICH location) and clinical severity scores (e.g., admission Glasgow Coma Scale). We acknowledge that these are established core predictors for secondary events after ICH. For instance, recent evidence strongly emphasizes that the baseline ICH volume itself is a predominant independent predictor of poor functional status, sometimes overshadowing other secondary intraventricular complications (28). Consequently, our biomarker-driven model (heavily relying on systemic inflammatory and coagulation indices like SII and D-dimer) is not intended to replace classic neuroimaging assessments. Rather, it serves as a supplementary, biochemical early-warning tool. It may be particularly valuable in identifying the severity of the systemic ‘immunothrombosis’ cascade and providing risk stratification in scenarios where immediate advanced neuroimaging is delayed.

Third, while we propose a “risk-stratified bridging strategy” involving tirofiban, this recommendation is theoretical and based on extrapolation from ischemic stroke trials. The safety and efficacy of antiplatelet therapy in the acute phase of ICH require confirmation through dedicated randomized controlled trials (RCTs). Finally, as an observational study, our findings establish association but not causality between immuno-thrombosis markers and RPIL, warranting further mechanistic investigation.

Furthermore, while the initial use of median imputation for missing clinical variables could theoretically constrain data variance—a common challenge in retrospective cohorts—our extensive sensitivity analysis utilizing the MICE method solidly confirmed that the XGBoost model maintained highly stable and robust predictive efficacy (mean AUC = 0.8358 ± 0.0054), thus effectively dispelling concerns regarding imputation bias.

Finally, strictly adhering to the newly published TRIPOD+AI consensus statement for machine learning in medicine, future efforts must prioritize prospective, multi-center external validation of these predictive models to rigorously ascertain their geographical and demographic generalizability prior to broad clinical implementation (15, 29).

## 5 Conclusion

In conclusion, this study represents the first large-scale application of machine learning to elucidate the “hemorrhage-ischemia paradox” in ICH patients. We identified a distinct high-risk phenotype characterized by systemic inflammation (high SII), metabolic stress (hyperglycemia), and hypercoagulability (high fibrinogen), providing strong clinical evidence for the immuno-thrombosis mechanism. Among 15 algorithms, XGBoost emerged as the optimal model for precision risk stratification, while our simplified nomogram offers a practical tool for bedside decision-making. These findings support a paradigm shift towards vigilant monitoring and potential risk-stratified antithrombotic interventions, paving the way for personalized management of secondary ischemic injury in ICH.

These findings support a paradigm shift towards vigilant monitoring and potential risk-stratified antithrombotic interventions, paving the way for personalized management of secondary ischemic injury in ICH. Nonetheless, prospective, multi-center external validation is required to definitively establish the generalizability and real-world clinical utility of these predictive tools.

## Data Availability

The original contributions presented in the study are included in the article/Supplementary material, further inquiries can be directed to the corresponding author.
